# Delayed Presentation of a Cervical Spinal Epidural Abscess of Dental Origin after a Fall in an Elderly Patient

**DOI:** 10.7759/cureus.621

**Published:** 2016-05-23

**Authors:** Alexa Bodman, Margaret Riordan, Lawrence S. Chin

**Affiliations:** 1 Department of Neurosurgery, SUNY Upstate Medical University

**Keywords:** spinal epidural abscess, mid-cervical spinal cord injury, delayed epidural hematoma

## Abstract

Spinal epidural abscesses are an uncommon cause of spinal cord injury but, depending on the size and presence of neurological deficits, urgent neurosurgical intervention may be required. We present a unique case of a patient presenting with a spinal epidural collection several days after a fall. While a spinal epidural hematoma was suspected based on the patient’s history and MRI findings, a spinal epidural abscess was found during surgery. The patient underwent laminectomy and instrumented fusion with successful treatment of her infection.

## Introduction

Spinal epidural abscesses (SEA) are an uncommon medical emergency with an incidence ranging from 0.2 - 1.2 of every 10,000 hospital admissions per year [1-2]. Pain with or without radiculopathy is often the initial symptom, and on average, patients present days to months after developing symptoms [3]. These symptoms are very difficult to distinguish from cervical spondylosis, which is much more prevalent. At the time of presentation, signs are variable with 39% of patients having paraplegia, 32% with fever, and 27% with urinary or bowel incontinence [4]. Magnetic resonance imaging (MRI) has been useful in improving the diagnosis of spinal epidural abscess and has contributed to the recent increased incidence of SEA [4-5]. After diagnosis, urgent treatment, including antibiotics and surgical decompression, may be necessary to facilitate neurologic recovery [6]. In some cases, the neurological deficit may not be reversible, particularly if there has been spinal cord infarction.

Common risk factors for developing spinal epidural abscesses include immunocompromising diseases, such as diabetes mellitus, end-stage renal disease, alcoholic liver cirrhosis, and intravenous drug use. Spinal trauma is a rare risk factor for the development of a spinal epidural abscess [3, 7-8]. Spinal epidural abscess after spinal fractures is thought to occur after the formation of a spinal epidural hematoma during the trauma, with the hematoma being a nidus for the infection [9]. The majority of spinal epidural abscesses related to trauma occur in the thoracic and lumbar spine [10]. Cervical spine abscesses after trauma are unusual and described only in case reports [11-12].

We present a unique case of a patient with a presumed delayed traumatic cervical spinal epidural hematoma revealed to be a spinal epidural abscess requiring surgical intervention.

## Case presentation

### History

A 77-year-old female presented to the Emergency Department with the loss of sensation in her bilateral lower extremities, followed by urinary and fecal incontinence, the loss of strength in both legs, and decreased strength in bilateral arms that began the prior evening. Her symptoms were preceded by six days of neck pain radiating into bilateral shoulders.

Ten days prior to this presentation, the patient had been to the emergency room after a fall from standing that resulted in a dental fracture of the anterior alveolar process, requiring dental splints. At the time of the fall, the patient had no neck pain and was neurologically intact on examination by Emergency Department physicians. In light of her unremarkable neck exam, she was discharged directly from the Emergency Department.

Her physical examination on initial neurosurgical evaluation was significant for a temperature of 35° Celsius and no sensation to pain or light touch below the T4 dermatome. Motor strength was Grade 0/5 in bilateral lower extremities, Grade 2/5 in bilateral hand intrinsics, wrist extensors, and deltoids, and Grade 3/5 in bilateral triceps and biceps. No rectal tone was present. White blood cells count was 8,900 cells/µL (4,000-10,000 cells/µL), hemoglobin - 10.2 g/dL (13.5-18 g/dL), hematocrit - 30.4% (41-53%), and platelets of 237,000 cells/µL (150,000-400,000 cells/µL).

Informed patient consent was obtained for treatment.

### Radiographic findings

**Figure 1:**
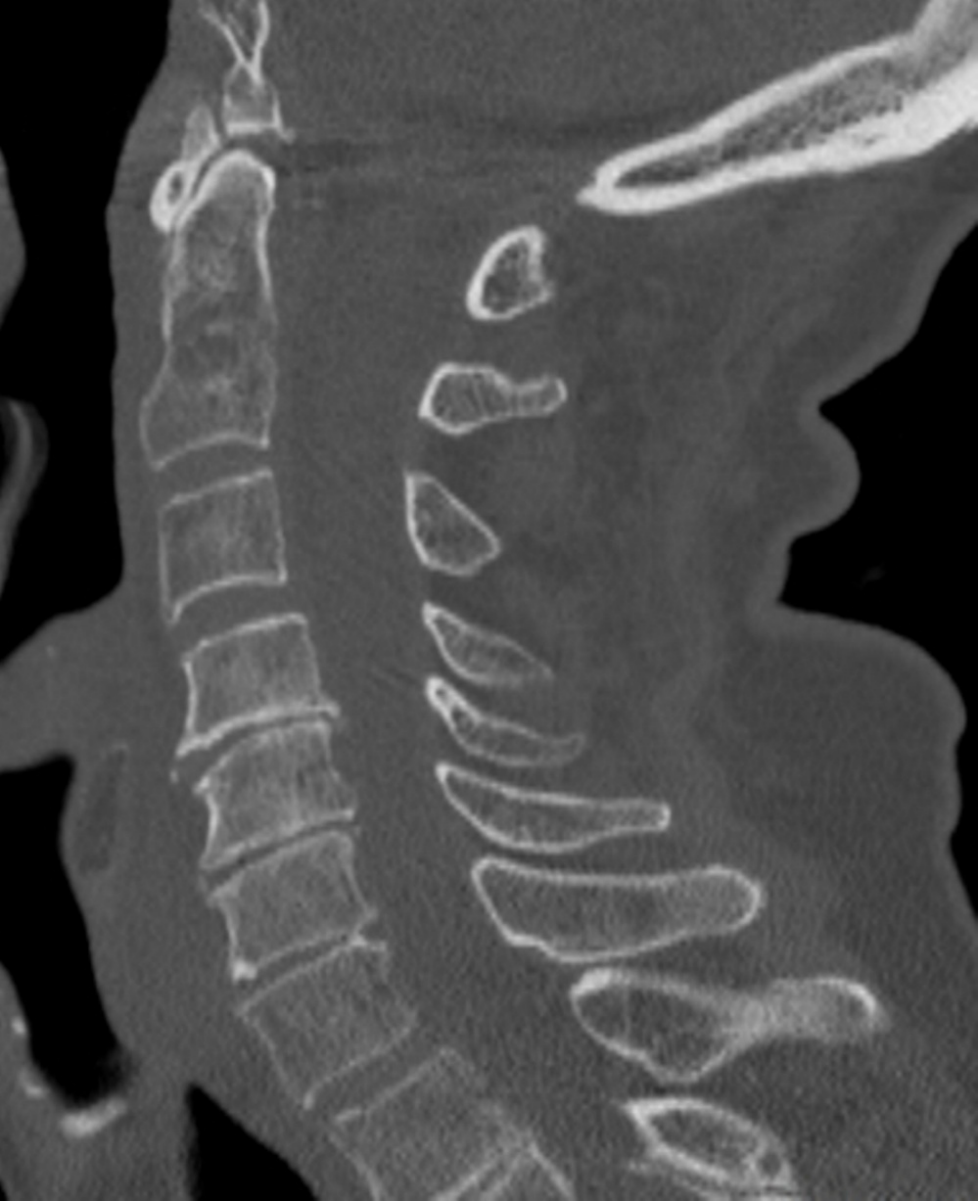
Sagittal CT of the Cervical Spine Sagittal CT image of the cervical spine showing no obvious fracture or dislocation at the time of the initial fall and evaluation by Emergency Department.

**Figure 2:**
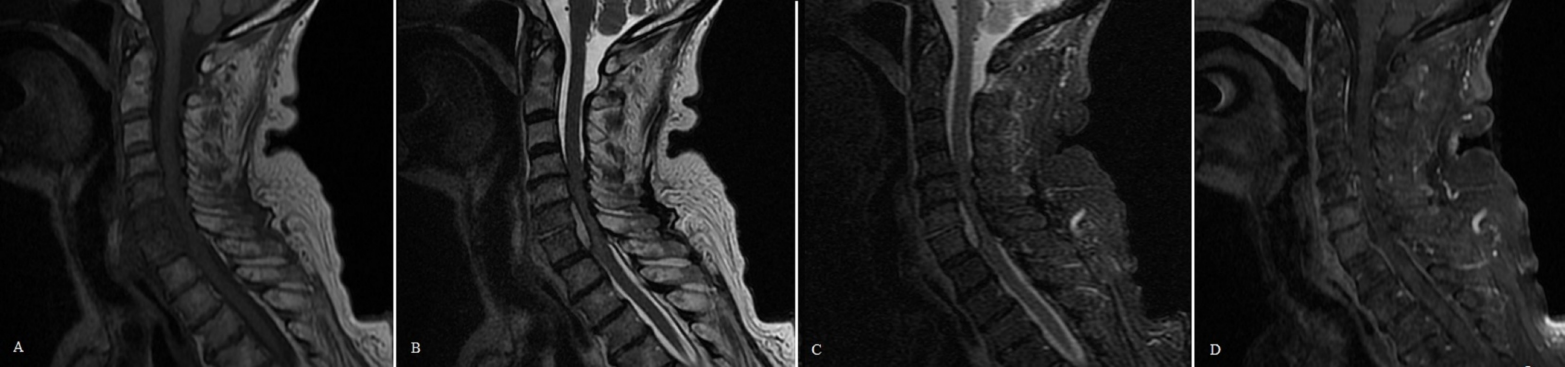
Sagittal MRI of Cervical Spine at Presentation A) T1-weighted images showing isointense epidural collection at the level of C6/7 and hypointense vertebral bodies of C6 and C7. B) T2-weighted images showing a hypointense epidural collection at the level of C6/7 concerning for hematoma with a slight hyperintensity of C6 and the C6 vertebral bodies. C) Short T1 inversion recovery (STIR) sequences images showing prevertebral edema and disruption of the anterior longitudinal ligament at C6/7 and slight hyperintensity of C6/7 disc space and C6 and C7 vertebral bodies likely representing bone marrow edema concerning for underlying injury though no discrete fracture line is noted. D) T1-weighted image with contrast; no significant ring enhancement of the collection is noted.

A computed tomography (CT) scan of the cervical spine was performed, which showed mild multilevel degenerative disease without acute findings (Figure 1). An MRI of the cervical spine with and without contrast was performed, the contrast being given due to her history of breast cancer, which showed epidural collections at the C6/7 level. Bone marrow edema in the C6 and C7 vertebrae was concerning for a non-displaced fracture, and disruption of the anterior longitudinal ligament with prevertebral soft tissue edema was also seen at C6/7. Given a minimal enhancement of the collection and the patient’s history of trauma and lack of constitutional signs, the MRI was most consistent with an epidural hematoma and a low suspicion for an epidural abscess (Figure 2). At this point, it was thought that the patient was presenting with a delayed epidural hematoma requiring emergent surgical evacuation.

### Operation

**Figure 3:**
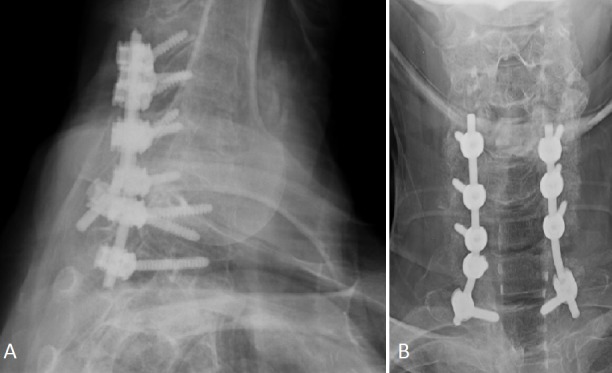
X-rays of Cervical Spine A) Lateral X-ray; B) AP X-ray showing postoperative images of instrumentation.

Posterior cervical laminectomies were performed extending from C4 to T1 with partial facetectomies. With gentle retraction on the thecal sac, the epidural collection was drained. Frankly purulent fluid was noted and culture swabs were sent for routine cultures and speciation. Instrumented fusion was then performed from C4 to T1 (Figure 3).

### Postoperative course

The patient was admitted to the Neuroscience Intensive Care Unit postoperatively and started on broad-spectrum antibiotics. C-reactive protein (CRP) and erythrocyte sedimentation rate (ESR) were drawn after the procedure and found to be elevated at 256.8 mg/L (1.0-3.0 mg/L) and 71 mm/hr (< 15 mm/hr), respectively. Cultures from the abscess taken during the operation grew *Streptococcus anginosus* susceptible to penicillin, ceftriaxone, and vancomycin. Blood cultures taken after the operation and administration of broad-spectrum antibiotics showed no growth. As the patient had a penicillin allergy, the patient was placed on six weeks of ceftriaxone and managed by the Infectious Disease Service. At the completion of the antibiotic course, the CRP and ESR normalized.

Upon discharge to an acute rehabilitation facility, the patient continued to have complete loss of motor strength and sensation in bilateral lower extremities; however, she had improved strength of the bilateral upper extremities with motor Grade 4/5 in the left deltoids, triceps, biceps, wrist extensors, and hand intrinsics and Grade 3/5 in the right deltoids, triceps, biceps, wrist extensors, and hand intrinsics. At her one-year follow-up, the patient’s upper extremity exam recovered almost completely with Grade 5/5 motor strength in bilateral deltoids, biceps, triceps, wrist extensors, and right hand intrinsics. The left hand intrinsics remained weaker at Grade 4/5. She continued to require a wheelchair but had also shown some improvement in bilateral lower extremities with Grade 3/5 motor strength. The patient continued to have a neurogenic bowel and bladder.

## Discussion

Spinal epidural collections are frequently treated with surgical decompression with or without spinal fusion [13]. Both spinal epidural abscesses and hematomas are similar in clinical presentation, and their distinction by MRI can be also difficult [14]. A confounding factor was a neck injury that was initially unrecognized because she had little direct cervical symptoms with her acute injuries coming primarily from dental trauma [15]. In this case, the patient’s recent history of trauma, lack of fever, leukocytosis, or other signs of infection and the MRI findings, suggested an epidural hematoma, but surprisingly, an SEA was found in surgery.

Spinal epidural abscesses are rarely described following spine injury. In some cases, the formation of an abscess may result from direct contamination of a fracture. For example, in one case report, a patient developed a spinal epidural abscess in association with an esophageal perforation after a thoracic compression fracture and traumatic cervical disc herniations that were treated operatively [16]. Another case reported a fatal spinal epidural abscess related to a hypopharyngeal perforation and C5 fracture after a motorcycle accident [11]. Unlike our case, these case reports describe a directly contaminated fracture that resulted in a localized infection.

Most cases of abscess formation after an injury are attributed to bacteremia. A case series of spinal epidural abscesses focusing on their bacterial etiology associated six of the cases, all in the thoracic and lumbar spine, with mild traumas. The series did not discuss the time course between the trauma and development of the abscess. The authors postulated that mild trauma may create a site susceptible to bacteremia [10]. In this case report, the patient probably suffered minor neck trauma that led to increased blood flow and bacterial seeding of mouth flora through the dental fractures to the cervical region. Unfortunately, as an abscess was not initially suspected, blood cultures were not taken until after broad-spectrum antibiotics had been administered. Therefore, although bacteremia was suspected, it was not confirmed by culture. In another case similar to ours, an elderly woman developed increasing neck pain after a fall, and cervical osteomyelitis with an associated spinal epidural abscess was discovered and treated conservatively with antibiotics. This patient had a known history of infective endocarditis that increased her risk of developing an abscess as well as mild immunosuppression from diabetes mellitus and systemic lupus erythematosus [12].

Spinal epidural hematoma formation may increase the risk of abscess formation as the hematoma creates an environment suitable for infection [9]. Delayed presentation of a spinal epidural hematoma after trauma is unusual but may start as back pain with radicular symptoms and then progress to more significant symptoms, such as numbness and paresis [17]. Cuenca, et al. reported a case of spinal epidural hematoma presenting with back pain followed by paralysis of both lower extremities ten days after an initial blunt spinal injury incurred during a motor vehicle accident. The spinal epidural hematoma was treated conservatively with steroids and patient had complete improvement [18]. Kang, et al. reported the development of a spinal epidural hematoma twelve weeks after a thoracic compression fracture related to osteoporosis [19]. Cervical epidural hematoma, requiring evacuation and decompression, has been reported to occur up to a week after the trauma [20].

In this case presentation, the patient may have had an epidural hematoma after her initial fall that served as a nidus for abscess formation. Her dental fractures likely resulted in bacteremia that seeded her area of injury. Dental fractures have not been previously reported to increase the risk of spinal epidural abscess formation, but other odontogenic etiologies of spinal epidural abscess have been reported. Dental extractions are a rare cause of cervical discitis and epidural abscess [21-22]. *Streptococcus milleri* and *Corynebacteria*, known bacteria to the mouth, as in our case, have been identified as the causative organisms in previous SEAs related to dental sources [21, 23]. *Staphylococcus aureus* and *Pseudomonas aeruginosa* have also been found in SEA related to an odontogenic source [24-25]. Case reports have shown that the diagnosis is frequently delayed up to several weeks [23-25].

In our case, instrumented fusion was planned prior to the start of the operation as the patient had suffered a recent neck injury and a significant portion of the facet needed to be removed in order to drain the anterior collection. Even after purulence was found, we proceeded with instrumentation because of concerns over potential instability with the trauma, loss of bone, and ligament integrity. Bydon, et al. performed a retrospective study that did not show an increased risk of infection with instrumentation in the setting of spinal epidural abscess [13]. These results were consistent with previous reports [26-27].

In conclusion, we present a case of a spinal epidural abscess masquerading as a delayed epidural hematoma in occult spinal trauma. Surgical decompression allowed us to make the proper diagnosis and the use of instrumentation did not lead to an adverse outcome.

## Conclusions

In conclusion, we present a case of a spinal epidural abscess masquerading as a delayed epidural hematoma in occult spinal trauma. Surgical decompression allowed us to make the proper diagnosis, and the use of instrumentation did not lead to an adverse outcome.
